# Human papilloma virus inoculation: why only girls?

**DOI:** 10.3747/co.v16i4.433

**Published:** 2009-08

**Authors:** L.Z.G. Touyz

Human papilloma viruses (hpvs) are causally associated with genital warts, papillomata, and oral squamous cell carcinoma (scc) 1,2. oral scc, with or without keratosis, is the sixth-most prevalent cancer. Non-keratotic scc derives from transmission of hpv 16. uterine cervical cancer is also causally associated with hpv infection and strongly associated with hpv 16 and hpv 18. Transmission may occur during sexual intercourse, orogenital sexual contact, and saviolum kissing (when mutual insertion of tongues allows for saliva exchange with all of the associated biologic and microbial load) 2,3.

Functionally, high-risk hpv infection contributes oncogenes E6 and E7, and 40 variants of E6 and E7 are related to speed of progression in the development of scc. The transcription factor nuclear factor κB (found in many tumours) and its activation pathway are targeted by the viruses 3,4.

Gardasil (Merck Frosst Canada, Kirkland, QC) is a recombinant quadrivalent intramuscular injection vaccine derived from hpv types 6, 11, 16, and 18 5 that is approximately 90% effective at producing immunity against hpv infection and preventing hpv from causing scc in females.

Given that young, sexually active males are reservoir carriers and a major source of hpv infection and transmission, not only should young females be inoculated before commencing sexual activity, but young males should be similarly targeted with a preventive strategy.
